# Recent advances in preclinical studies combining hyperthermia therapy with novel immune checkpoint targeting agents

**DOI:** 10.3389/fimmu.2026.1722115

**Published:** 2026-03-23

**Authors:** Na Li, Jian Yu, Ruoyu Wang, Guanyu Gong

**Affiliations:** 1 Laboratory of Oncology, Affiliated Zhongshan Hospital of Dalian University, Dalian, Liaoning, China; 2 Liaoning Provincial Key Laboratory for Brachtherapy and Hyperthermia Therapy, Dalian University, Dalian, Liaoning, China; 3 Hyperthermia Therapy Center, Affiliated Zhongshan Hospital of Dalian University, Dalian, Liaoning, China; 4 Department of Medical Oncology, Affiliated Zhongshan Hospital of Dalian University, Dalian, Liaoning, China

**Keywords:** cancer treatment, hyperthermia, immune checkpoint molecules, immune co-stimulatory molecules, immunotherapy, tumor microenvironments

## Abstract

Hyperthermia has been used as an adjuvant therapy alongside radiotherapy and chemotherapy for cancer treatment in some countries. However, since the 2000s, growing evidence has indicated that hyperthermia exerts regulatory effects not only on cancer cells but also on stromal immune cells and the research interest in this topic has grown notably in the current “era of immunotherapy”. Of particular interest to oncoimmunologists and hyperthermia researchers, recent studies have shown that hyperthermia modulates the expression of a wide range of immune checkpoint and co-stimulatory molecules. In addition to the PD-1/PD-L1 and CTLA-4/CD80/CD86 checkpoints previously reported and intensively discussed in existing reviews, recent studies indicate that hyperthermia exerts a broader regulatory effect on many other checkpoint and co-stimulatory molecules, include TIGIT/CD155, Tim-3/Gal-9, OX40/OX40L, and 4-1BB/4-1BBL on T cells, CD47/SIRPα on macrophages, and CD40/CD40L on dendritic cells. The present review aims to provide a complementary update, focusing specifically on recent advances in understanding how hyperthermia regulates the expression of these newer targets, as well as preclinical evidence for combining hyperthermia with novel therapeutic agents targeting these molecules. The insights gained from these preclinical studies could serve as a valuable foundation for future experimental investigations and clinical translation.

## 1 Introduction

According to the recent epidemiological statistics, there were nearly 20 million newly diagnosed cancer cases globally in 2022, with approximately 9.7 million deaths associated with cancer (1). Given the persistently high mortality rate of cancer, there remains a constant need to explore novel methods and combination strategies aiming at enhancing therapeutic efficacy, reducing treatment-related adverse effects, improving quality of life, and prolonging overall survival for patients (2).

Hyperthermia therapy, a physiotherapy approach for combating cancer, has been approved in China and some European countries to be used as an adjunctive therapy to enhance tumor sensitivity to chemotherapy and radiotherapy (3). As time elapsed, accumulating evidence has indicated that hyperthermia therapy exerts multiple additional effects on cancer tissues. Most importantly, hyperthermia has been shown to regulate multiple immune signaling pathways that mediate interactions between cancer and immune cells, leading to a substantial reshaping of the tumor microenvironment and such topic has been extensively reviewed in previous literature (4–6). Although currently validated only in animal models, hyperthermia is increasingly recognized as a potential adjunctive therapy that could enhance therapeutic efficacy of both established and emerging immune checkpoint-targeted agents (7, 8).

In the current “era of immunotherapy”, the success of anti-PD-1/PD-L1 and anti-CTLA-4 immune checkpoint inhibitors (ICIs) has inspired pharmaceutical companies and research groups to explore next-generation therapeutic agents targeting additional inhibitory and activating immune checkpoints (9, 10). While the overall response rate of current immune checkpoint inhibitors (ICIs) remains relatively low, ranging from 10% to 50%, they are also associated with non-negligible adverse effects (11, 12). Combination therapy using two different ICIs has been shown to achieve higher response rates, but is meanwhile associated with more severe immune-related adverse events (13, 14). Combination therapies involving ICIs with chemotherapy or hypo-/hyper-fractionated radiotherapy have been actively investigated in clinical trials, demonstrating improved response rates to varying degrees (15, 16).

Hyperthermia therapy is known for its favorable safety profile and is rarely associated with severe adverse effects (17, 18). If feasible, hyperthermia could be a suitable candidate for combination therapy with immune checkpoint inhibitors (ICIs). The present review aims to update our understanding of the potential synergistic effects of hyperthermia with emerging immune checkpoint-targeted agents and to elucidate the potential mechanism associated with hyperthermia’s immunomodulatory effect. To this end, we searched the PubMed website using the terms “hyperthermia OR thermal therapy OR thermal ablation OR photothermal therapy” AND “immune checkpoints OR TIGIT OR Tim-3 OR CD40 OR OX40 OR 4-1BB OR CD47” during the years between 2000 and 2025. The literature were manually examined for their relevance to the topic of our interest. The insights gained from these preclinical studies could serve as a valuable foundation for future experimental investigations and clinical studies.

## 2 Inhibitory immune checkpoints

The immune checkpoints, as receptors/ligands expressed on the cell surface, regulate the communication among T cells, antigen presentation cells and tumor cells. The immune checkpoints fine-tune the immune response towards the pathogenic targets (e.g. microbes in infection and tumor cells). The inhibitory checkpoints, including PD-1/PD-L1, CTLA-4/CD80/CD86, TIGIT/CD155, Tim-3/Galectin-9, CD47/SIRPα, LAG3/MHC, negatively regulate the activity of immune cells to avoid excessive response such as “cytokine storm”. However, cancer cells take advantage of such mechanism to evade the host immune system and therapeutic agents blocking the function of these inhibitory immune checkpoints has been shown to re-boost the anti-tumor immunity. Hyperthermia therapy, on the other hand, has been shown to regulate the expression of various inhibitory checkpoints, including the newer generation TIGIT/CD155, Tim-3/Galectin-9 and CD47/SIRPα molecules, which findings are of particular interest to us. We summarize the studies integrating hyperthermia with therapeutics targeting these checkpoints in the below sections.

### 2.1 TIGIT

TIGIT is an inhibitory receptor expressed on T cells (including CD4+ T cells, CD8+ T cells and T-reg cells) and NK cells (19). It competes with CD226 (DNAM-1), which is also expressed on T cells, for binding with two ligands, CD155 (PVR) and CD112 (PVRL2, nectin-2), which are expressed on tumor cells and antigen-presenting cells (20, 21). The binding of CD155-TIGIT inhibits and the binding of CD155-CD226 promotes the functional activation of T cells and NK cells (22). TIGIT is frequently co-expressed with PD-1 as co-inhibitory molecules on CD8+T cells, including CD8+ tumor-infiltrating lymphocytes (TILs) in the microenvironment, in both mouse and human tissues (23). Because TIGIT is abnormally upregulated in multiple types of cancer (24), it is currently studied as an emerging target for combination immunotherapy with anti-PD-1 inhibitor and with other therapeutic approaches (23).

Combination treatment using hyperthermia and anti-TIGIT mAb has been investigated in two studies using preclinical models. Chen Y et al. applied microwave ablation (MWA, heating at 70 °C for 2 to 4 minutes) to MC38 subcutaneous murine model of colon cancer (25). Before treatment, the authors observed that TIGIT expression is higher in TILs than in splenocytes and suggested that TILs might be the main type of immune cell responding to anti-TIGIT therapy. The authors applied microwave hyperthermia to ablate the tumor on the treatment side and ten days later, they observed increased TIGIT expression in TILs of the tumor on the abscopal (non-ablated) side. The increased TIGIT expression may be caused by systemic response to tumor ablation that released tumor-specific antigens. In combination therapy using MVA and anti-TIGIT mAb, tumor growth was found to be suppressed on both treatment and abscopal sides. Immunologically, the authors observed increased number of CD45+ and CD8+ T cells in the MWA group and TIGIT group, and increased number of CD45+, CD4+ and CD8+ TILs and NK cells in the MWA+anti-TIGIT group, in which group the CD8+ T cells also exhibited higher expressions of GZMB, IFN-γ and TNF-α cytokines. Their single-cell RNA sequencing results suggested that MWA therapy mobilized T cell population and anti-TIGIT mAb prevented CD8+ T cell exhaustion. Therefore, it appears that microwave ablation by itself increases number of CD8+ T cells but meanwhile upregulates TIGIT expression, whereas anti-TIGIT copes with the problem of TIGIT upregulation, and the two approaches harmonize with each other.

Another study by Wang X et al. employed mesoporous SiO2 and zirconium diboride (ZrB_2_)-based nanomaterials to conduct photothermal therapy (PTT) on 4T1 subcutaneous model of breast cancer (26). The temperature increase was 23 °C upon near-infrared (NIR) light exposure *in vitro* and was found to cause immunogenic cell death (ICD) with elevated calreticulin expression, ATP secretion and HMGB1 relocation. The authors further incorporated TIGIT and PD-1 dual-specificity checkpoint inhibitor into this nanomaterial. *In vivo* application of this nanomaterial to 4T1 tumors resulted in a temperature rising to 55.6 °C and significantly inhibited tumor growth and distal metastasis. Immunological analysis showed increased maturation of dendritic cells in the draining lymph nodes, increased number of CD4+ and CD8+ T cells and decreased number of T-regs in spleen, and increased number of CD4+ and CD8+ T cells (with higher expression level of IFN-γ and TNF-α) in TILs, in the PTT + dual specific TIGIT/PD-1 blockade group comparing with the other groups. suggesting synergistic combination of PTT-induced ICD and dual TIGIT and PD-1 blockade.

So far, no evidence has shown that thermal stress *per se* directly modulates TIGIT expression in T cells. Yet one study suggested that TIGIT expression in melanoma cells and T cells was regulated by DNA methylation, an epigenetic process known to respond to environment changes (27), and thus it is possible that TIGIT expression could be altered after thermal therapy. It is more likely that hyperthermia promotes systemic immune response after ICD induction and changes the expression of checkpoint molecules (25, 26). Hyperthermia treatment has been shown to increase the release of HMGB1 (28, 29), calreticulin (30, 31), ATP (32, 33) and various heat shock proteins (34, 35), which all participate to enhance anti-tumor immune response (6, 36). Hyperthermia has also been suggested to enhance IL-2 secretion in CD4+ T helper cells, a master cytokine responsible for promoting the differentiation of naive T cells and proliferation of functionalized T cells (37–39). These are additional underlying mechanisms of the synergistic effect between hyperthermia and anti-TIGIT treatment. Because the studies by Chen Y et al. and Wang X et al. used high temperature (70 °C and 55.6 °C, respectively), it is still unknown whether the microwave/radiofrequency hyperthermia performed in clinics could similarly synergize with anti-TIGIT therapy.

### 2.2 Tim-3

Tim-3, alternatively known as HAVCR2, is another inhibitory checkpoint receptor naturally expressed on T cells (e.g. CD4+ T cells, CD8+ T cells and T-reg cells) and also on innate immune cells (dendritic cells, monocytes, macrophages and NK cells) (40). Tim-3’s major binding ligand is Galectin-9 (Gal-9) but it can also bind with other ligands including CEACAM-1, HMGB1 and phosphatidyl-serine(PtdSer) (41–43). Gal-9 is one of the galectin family members and its overexpression has been found in a variety of tumors (44, 45). The binding of Tim-3 (T cells) with Galectin-9 (tumor cells) has been suggested by various studies to induce T cell exhaustion (46), including CD4+ Th1/Th17 cells and CD8+ CTLs, and downregulate IFN-γ and TNF-α production (47, 48). The binding of Tim-3 (T cells) with CEACAM-1 (other T cells) has been suggested to inhibit T cell activation (49). The binding of Tim-3 (dendritic cells) with extracellular HMGB1 released from dying cells has been suggested to impede the functional maturation of DCs (50). The binding of Tim-3 on phagocytes and NK cells with PtdSer, which is externalized from dying cancer cells, has been suggested to activate “eat-me” signal for phagocytosis but meanwhile repress inflammasome activation (51, 52) and suppress T cell (53) and NK cell activation (54). In overall, Tim-3 exerts a multifaceted role suppressing the activity of many types of immune cells.

Huang TY et al. evaluated the therapeutic potential of a liposome-based photothermal nanoparticle containing indocyanine green (ICG) in CT26 and MC38 (colon cancer) orthotopic tumors (55). Using this namomaterial, the temperature of tumors rise up to 57-60 °C upon NIR exposure but only minimal inhibition of tumor growth was observed. The number of Tim-3+ and PD1+Tim-3+ CD8+ T cells was found significantly upregulated in tissues treated with this PTT method. Combination of the ICG-mediated PTT, anti-PD1 mAb and anti-Tim-3 mAb was found to result in strong suppression of tumor growth, which effect was not observed in either monotherapy approach. Another study by Ren H et al. found that in the B16-F10 melanoma model, Galectin-9 is highly expressed in tumor cells and Tim-3 is highly expressed in infiltrating CTLs and NK cells (56). The authors designed a transdermal photothermal nanomaterial (FSGG) conjugated with Galectin-9 siRNA to treat the melanoma model. The tumor temperature was heated up to 45.4 °C upon NIR exposure. The authors observed suppression of tumor growth by this nanommaterial generating concurrent PTT and Tim-3-Gal-9 inhibition effect. The authors found increased IL-2, TNF-α and IFN-γ production and decreased T cell apoptosis in the PTT + Galectin-9 siRNA treatment group, but only increased IL-2 in the PTT monotherapy group. These results suggest that blockade of Tim-3-Gal-9 inhibitory checkpoint synergizes with PTT therapy by limiting T cell exhaustion.

**Figure 1:**
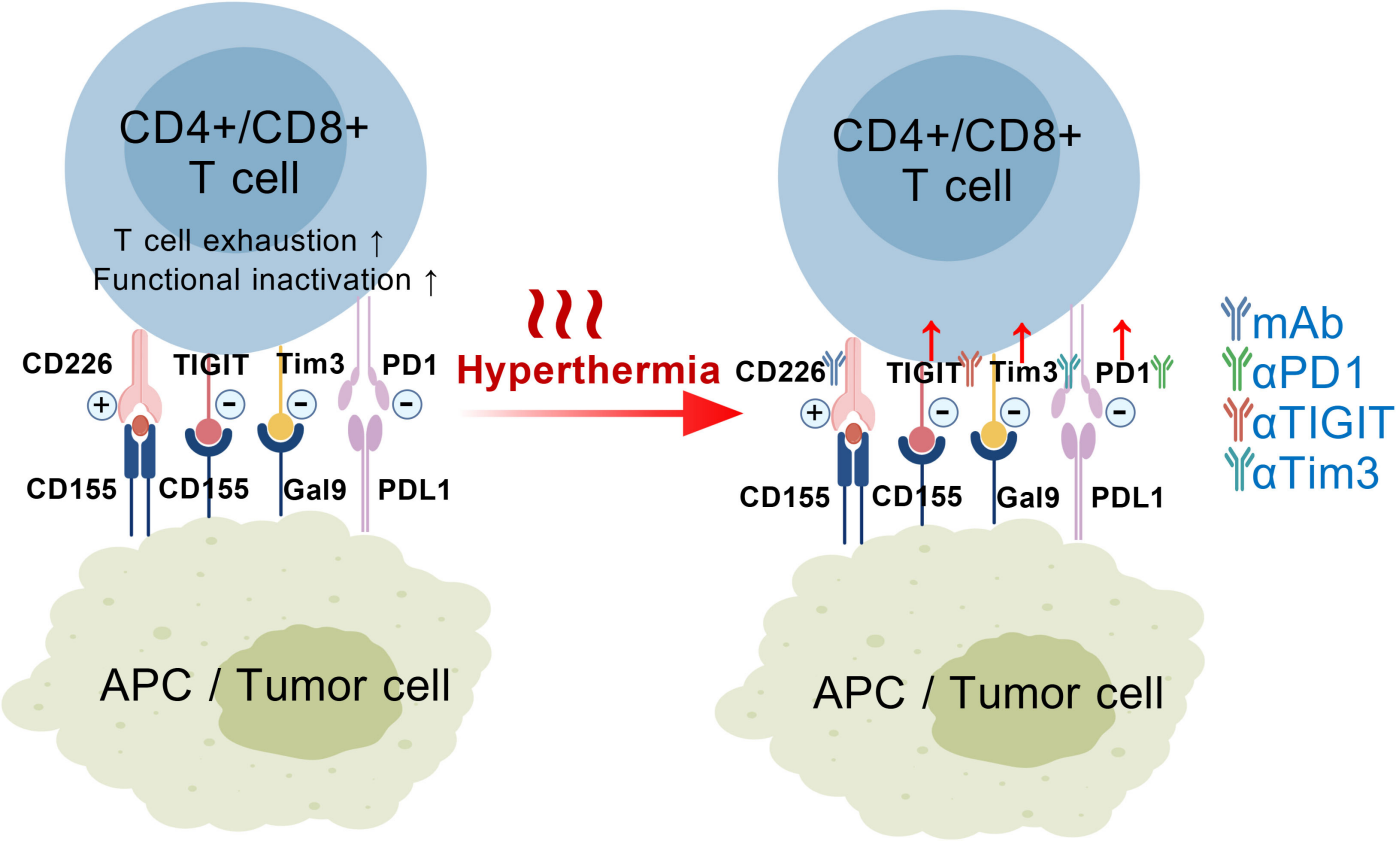
The interaction between CD4+/CD8+ T cells and antigen-presenting cells (APCs) or tumor cells is regulated by various immune checkpoints, such as the activating checkpoint CD226-CD155, and inhibitory checkpoints including TIGIT-CD155, Tim-3-Galectin-9, and PD1-PDL1. Activation of theseinhibitory checkpoints results in T cell exhaustion and/or functional impairment. Hyperthermia treatment has been shown to upregulate the expressionof TIGIT, Tim-3, and PD1 molecules on T cells, thereby enhancing their responsiveness to immune checkpoint blockade with monoclonal antibodies.

The regulatory effects of hyperthermia on TIGIT, Tim-3, and PD-1 expression are similar that the frequencies of TIGIT+, Tim-3+ and PD-1+ T cells dramatically increase following microwave ablation or photothermal therapy (25, 26, 36, 55–57). Increased expression of these inhibitory checkpoint molecules after hyperthermia generally represents immunosuppressive status, but when being coupled with mAb-based blocking treatment, the responsiveness becomes stronger. A schematic figure is provided (**Figure 1**) to illustrate the mechanistic linkage between hyperthermia treatment and mAb therapy targeting TIGIT/Tim-3/PD-1 inhibitory checkpoints.

### 2.3 CD47

CD47, elsewhere known as integrin-associated protein (IAP), is an inhibitory glycoprotein of the immunoglobulin superfamily and its function is to prevent the engulfment of cancer cells by phagocytes, particularly the macrophages (58). While the binding between phosphatidylserine and Tim-3 signifies “eat me” signal (51), CD47 on the surface of tumor cells binds with its partner signal-regulatory protein α (SIRPα) on the surface of phagocytes and signifies “don’t eat me” signal. The CD47-SIRPα binding activates the immunoreceptor tyrosine-based inhibitory motifs (ITIMs) of the intracellular proportion of the SIRPα receptor, ITIMs recruit Src homology region 2-domain-containing phosphatase 1 (SHP1) and SHP2, and SHP1/2 prevents the assembly of myosin at the phagocytic synapses to inhibit phagocytosis (59). Various studies have reported CD47 overexpression in non-small cell lung cancer, stomach cancer, colorectal adenocarcinoma, pancreatic cancer and hematological malignancies, and higher expression level of CD47 is associated with unfavorable clinical outcome (60). Targeting CD47 for cancer treatment is a hot-topic in clinical trial studies and preclinical animal model studies.

Because of the ectopic expression of CD47 on tumor cells, anti-CD47 mAb was used to coat various thermogenic nanoparticles to facilitate the engulfment of these particles into cells for cancer treatment. Wu CC et al. conjugated anti-CD47 mAb with pegylated silica-core gold nanoshells (pSGNs) and evidenced better therapeutic efficacy of anti-CD47-pSGNs than non-specific-Ig-pSGNs in the HIPEC treatment of peritoneal carcinomatosis modeled using CD47-overexpressing TOV21G ovarian cancer cells (61). Rezaei G et al. conjugated anti-CD47 single-chain variable fragment (scFv) with magnetic nanoparticles (MNPs) and demonstrated increased tumoricidal efficacy in CD47-high EJ138 and 5637 bladder cancer cells (62). Similarly, a recent study by Ji X et al. generated a SIRPα-overexpressing RAW264.7 cell line carrying microwave-responsive Prussian blue nanoparticles (nanoPB) (63). The SIRPα-coated nanoPB-incorporated macrophages recognize CD47-overexpressing K7M2 osteosarcoma cells, heat tumor tissue up to 50 °C upon microwave induction and suppress tumor growth. Tissue analysis revealed increased iNOS/CD206 (M1/M2) ratio, increased CD8+ T cell infiltration and higher proinflammatory TNFα and IL-17 cytokine production, indicating enhanced macrophage phagocytosis and M1-mediated T cell response.

Hyperthermia therapy has been shown to reduce the expressions of CD47 and SIRPα in cancer cells. Mouratidis PXE et al. observed downregulated CD47 in HCT116 and HT29 colon cancer cells after exposure to thermal therapy at a dosage of 60-120CEM_43_ (64). Adkins I et al. reported that high temperature (> 43 °C) rather than low temperature (<42 °C) induces decreased CD47 expression (65). Wang S et al. reported that ferrimagnetic vortex-domain iron oxide (FVIO) nanoparticle-mediated temperature increase by 24 °C resulted in decreased CD47 expression in Hep1–6 mouse hepatic cancer cells and decreased SIRPα expression in RAW264.7 macrophages (66).

Hyperthermia therapy has also been shown to promote M1 polarization. For example, Wang S et al’s study demonstrated that hyperthermia increased M1 polarization and phagocytic activity of RAW cells. Their magnetic hyperthermia at 43 °C were found to inhibit growth of subcutaneous H22 tumors and tissue analysis revealed increased M1 polarization, increased CD4+ and CD8+ T cell population, decreased T-reg population, and inhibited CD47-SIRPα signaling in the hyperthermia treatment group. Chang M et al. synthesized nanoparticles containing Cu2O@CaCO3@HA that would be converted into photosensitive Cu_31_S_16_ nanocrystals in acidic tumor environment (67). The authors found that the nanoparticle increased tumor temperature to ~50 °C and increased ROS production upon NIR exposure, and efficiently promoted the macrophages to switch from M2 polarization to M1 polarization (decreased CD206+ and increased CD86+ macrophages) (67, 68). The Cu2O@CaCO3@HA nanoparticles (with NIR) efficiently sensitized subcutaneous CT26 tumors to anti-CD47 mAb therapy by upregulating the M1 population, increasing IL-12 production, decreasing IL-10 production, and promoting infiltration of CD8+ and CD4+ T cells into tumors (67). Together, these studies suggest that hyperthermia promotes M1 polarization which might further increase the efficacy of anti-CD47 therapy.

**Figure 2:**
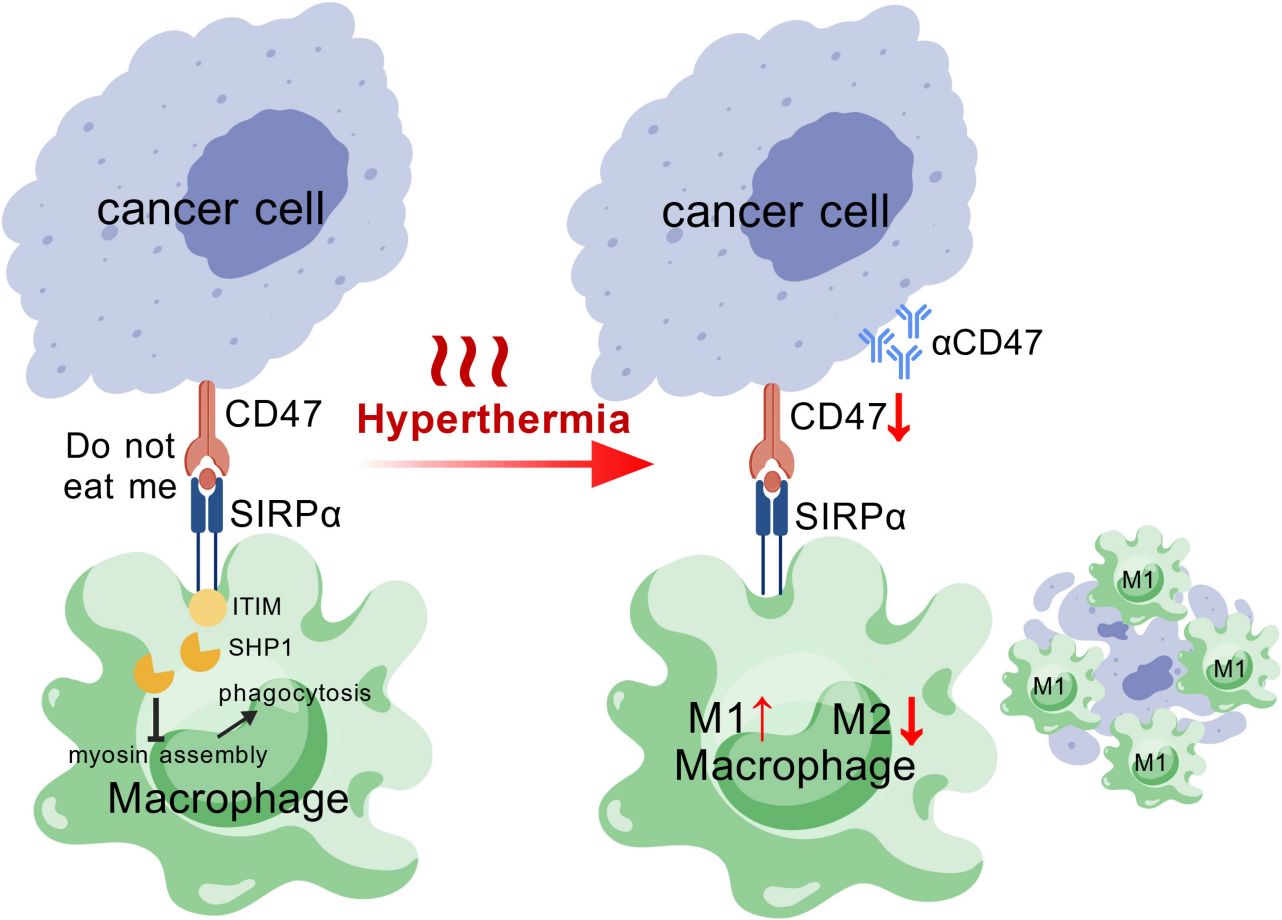
The interaction between cancer cells and macrophages is regulated by the CD47–SIRPα immune checkpoint, which transmits a “do not eat me” signal. Upon binding, the intracellular ITIM motif of the SIRPα receptor recruits SHP1, which further inhibits myosin assembly, thereby suppressing macrophage phagocytosis and preventing the engulfment of cancer cells. Hyperthermia treatment has been shown to downregulate CD47 expression on tumor cells, thereby synergizing with anti-CD47 monoclonal antibody therapy. In addition, hyperthermia is suggested to promote M1 (phagocytically active) macrophage polarization.

Macrophages are the most abundant type of immune cells in cancer microenvironments. Enhancing the phagocytotic activity of macrophages by targeting the CD47-SIRPα axis represents a novel strategy of cancer immunotherapy. Hyperthermia exerts multiple beneficial effects on enhancing macrophage phagocytosis, by downregulating the expression of CD47/SIRPα molecules (64–66), promoting ICD of tumor cells for phagocytes to engulf (63, 66, 67), and enhancing M1 polarization for stronger phagocytotic activity and proinflammatory cytokine release in the TiME (63, 66–68). As a result, hyperthermia has potential to synergize with anti-CD47 therapy and a schematic diagram is provided in **Figure 2** to explain the underlying mechanisms.

In addition to the impact on macrophages, CD47 blockade has been reported to enhance the activity of dendritic cells (69). This is because that DCs express SIRPα and CD47-SIRPα checkpoint impedes DCs’ antigen presentation function. CD47 mAb was found to re-activate the cGAS-STING pathway to prime T cells to eliminate the immunogenic tumors (69). Hyperthermia has also been known to activate DCs through a variety of other mechanisms (36, 70). Therefore, it remains necessary to examine the synergistic effect between hyperthermia and anti-CD47 therapy from the view of dendritic cell-based mechanisms.

## 3 Excitatory immune checkpoints

The excitatory immune checkpoints, also known as activating or co-stimulatory molecules, are receptors/ligands expressed on the cell surface that positively regulate the activity of antigen presentation cells and T cells. These co-stimulatory molecules, including CD40/CD40L, OX40/OX40L, 4-1BB/4-1BBL, ICOS/ICOSL, CD80/CD28 and etc, are an important part of positive feedback loop for full immune activation. However, in cancer microenvironment, these excitatory immune checkpoints are often under-expressed or inhibited. Hyperthermia therapy has been evidenced to increase the expression of CD40, CD80, OX40 and 4-1BB molecules, generating potential synergistic effect with agonistic mAb therapies that stimulate these targets. We summarize the studies of hyperthermia in relation with therapeutics targeting these excitatory checkpoints below.

### 3.1 CD40

CD40 is a co-stimulatory molecule expressed on the surface of antigen presentation cells including dendritic cells, macrophages, B cells and fibroblasts (71). Ectopic CD40 expression has also been reported in malignant cells such as lymphoma, melanoma, and prostate, lung, nasopharynx, bladder, cervix and ovary cancers (71, 72). Its counterpart CD40L is an inducible ligand/receptor expressed on the surface of activated T, B and NK immune cells, mast cells, macrophages and monocytes under inflammatory condition (73). The binding of CD40-CD40L would license DCs to mature to trigger CD8+ T-cell priming and promote continuous differentiation and clonal expansion of CD4+ T cells, which amplifies the inflammatory signaling (72, 74). Owing to its critical role in regulation of antigen presentation and T cell maturation, research interest in targeting CD40-CD40L axis is high.

Hyperthermia therapy has been reported to positively regulate CD40 expression in dendritic cells. In several previous studies, human and/or murine-derived DCs were exposed to 39-41 °C hyperthermia and these DCs showed upregulated CD40, CD80 and CD86 expression, increased production of proinflammatory cytokines and enhanced interactive activity with CD4+ T cells (75–77). These authors observed unchanged CD40 expression after silencing HSF1 gene or addition of HSP90 inhibitors, believing that heat shock response was responsible for CD40 upregulation (76, 77). While it is also known that HSPs facilitate the uptake of tumor antigens by DCs thorough formation of HSP-Ag complex (78, 79) and heat stress enhances the release of proinflammatory cytokines (79), it is also possible that hyperthermia indirectly upregulates CD40 expression as a result of accelerated DC maturation.

**Figure 3:**
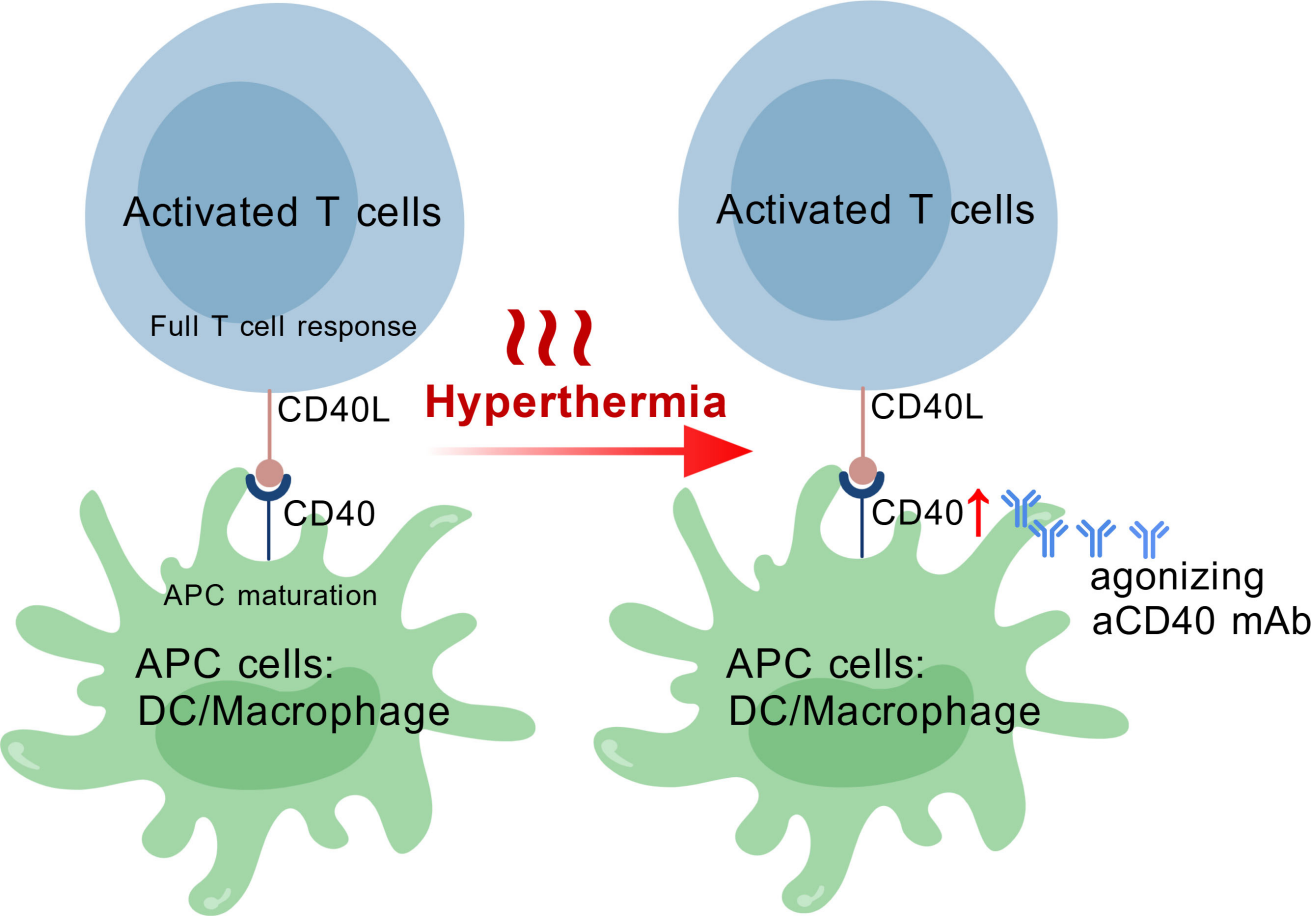
The co-stimulatory molecule CD40 is expressed on antigen presentation cells (APCs), including dendritic cells and macrophages, while its ligand CD40L is present on activated T cells. The binding of CD40 with CD40L promotes the maturation of APCs. Hyperthermia has been shown to upregulate CD40 expression on dendritic cells, thereby enhancing the therapeutic efficacy of agonistic anti-CD40 monoclonal antibodies.

So far there is one study exploring the combination of hyperthermia with agonistic anti-CD40 mAb. Singh MP et al. used focused ultrasound (FUS)-mediated hyperthermia at 42-45 °C and intratumoral injections of anti-CD40 mAb to treat subcutaneous B16F10 murine model of melanoma (80). The authors reported significantly improved tumor inhibition when combining the two approaches. Tissue analysis revealed increased infiltration of tumor-specific CD4+ and CD8+ T cells, increased IL-2 and IFN-γ production by CD4+ and CD8+ T cells, and increased M1 polarization in the FUS + anti-CD40 treatment group. These changes were not observed in FUS and anti-CD40 monotherapy groups. Their results suggest that hyperthermia and agonistic anti-CD40 mAb have a great potential to synergize with each other. The potential mechanism underlying the synergy between the two approaches is that hyperthermia promotes DC maturation and as a result, increases CD40 (and also CD80/CD86) expression on DCs and agonistic anti-CD40 mAb further accelerates the maturation process to trigger T cell mediated anti-tumor response. An explanatory diagram is provided in **Figure 3**.

### 3.2 OX40

OX40 is a T cell-specific costimulatory molecule that regulates the full activation of T cells (81, 82). OX40 is not expressed on naive T cells but after TCR engagement with antigen, its expression is upregulated on the surfaces of CD4+ T helper cells (including T-regs) and CD8+ T cells (81, 83). Weak OX40 expression has also been found in NK cells, NK T cells and neutrophils (82). Its ligand OX40L is expressed on antigen-presentation cells (DCs, macrophages and B cells) (81, 82). The binding of OX40-OX40L has been suggested to play a crucial role in survival and expansion of differentiated T cells and avoid of T cell exhaustion (83, 84).

Ectopic expression of OX40L has been reported in cancer cells under various conditions of therapeutic stress, including hyperthermia (85–87). Stoll E et al. observed upregulated OX40L expression by 2–3 folds in human glioblastoma U87 cells and by 1.3-1.5 fold in U251 cells after 39-44 °C hyperthermia treatment (86). They also found upregulated expression of some other immune checkpoint molecules including PD-L1, PD-L2, HVEM, ICOS-L, CD137-L and CD70 (86). The same research group also observed upregulated OX40L expression in breast cancer and hepatocellular carcinoma cell lines after hyperthermia (88, 89). The meaning of ectopic OX40L expression in cancer cells has not yet been understood but is likely to participate in immune evasion (90, 91).

Potential synergistic effect between hyperthermia therapy and agonistic anti-OX40 mAb has been observed in some preclinical mouse model studies. Mahmood J et al. treated subcutaneous Pan02 pancreatic tumors with thermal therapy (by immersing tumor in 42.5 °C water-bath) with radiotherapy (8 Gy X2) and anti-OX40 mAb (85). The authors observed dramatic growth inhibition by triple therapy, mild inhibition by RT+OX40 or Ht+OX40 therapy, but no inhibition by monotherapy. Tissue analysis suggested that OX40 agonist induced a boost of CD8+ and CD4+ T cell expansion. Interestingly, the number of CD4+ T cells was significantly decreased after RT and re-increased only after addition of anti-OX40 mAb (RT+OX40). The number of CD8+ T cells was also decreased by RT and re-increased after addition of either HT or anti-OX40. These findings suggested that hyperthermia therapy exerts beneficial effects preferentially on CD8+ T cells whereas OX40 agonist exerts beneficial effects on both CD4+ and CD8+ T cells.

Ni W et al. designed a photosensitive “MMH” nanomaterial and treated subcutaneous CT26 colon cancer tumors with MMH-mediated photothermal therapy (at 55 °C) in combination with anti-OX40 mAb (92). The authors found synergistic inhibition of tumor growth by combination treatment. Interestingly, comparing with the OX40 monotherapy group and the MMH + OX40 group, the MMH + PTT + OX40 group was found characteristic with significantly upregulated T cell response (increased CD3+, CD4+, CD8+ T cells and decreased T-regs), upregulated DC activation and downregulated infiltration of MDSCs in the TiME. Production of TNF-α, IL-6 and IFN-γ proinflammatory cytokines were also found to be dramatically increased in the MMH + PTT + OX40 group comparing with the other groups. Similarly, Chang X et al. used Ti3C2-MXene-Au (TMA)-based nanomaterial and combined PTT (at 55 °C) with anti-OX40 agonist to treat the 4T1 breast cancer tumors (93). The authors observed no tumor growth inhibition in the TMA/OX40/TMA+OX40 groups (without PTT), and strong inhibition in the TMA-PTT+OX40 group. FACS analysis reported increased numbers of CD44+ activating T cells, CD4+ and CD8+ T cells and decreased number of MDSCs in the TMA-PTT+OX40 group. The authors also found increased DC activation and M1/M2 polarization in this group. Momenzadeh N et al. designed photothermal cuttlefish ink-based nanoparticles (CINPs) that elevated tumor temperature to 40.7-46.6 °C and applied CINP-mediated PTT with αnti-OX40 mAb to treat 4T1 tumors (94). It was found that *in vitro* treatment of bone marrow-derived macrophages with PTT promoted M2 to M1 repolarization and *in vivo* treatment increased the population of OX40+ CD8+ T cells. Combined PTT + anti-OX40 treatment strongly inhibited tumor growth and caused dramatic increase of infiltrating CD8+ T cells in TiME.

**Figure 4:**
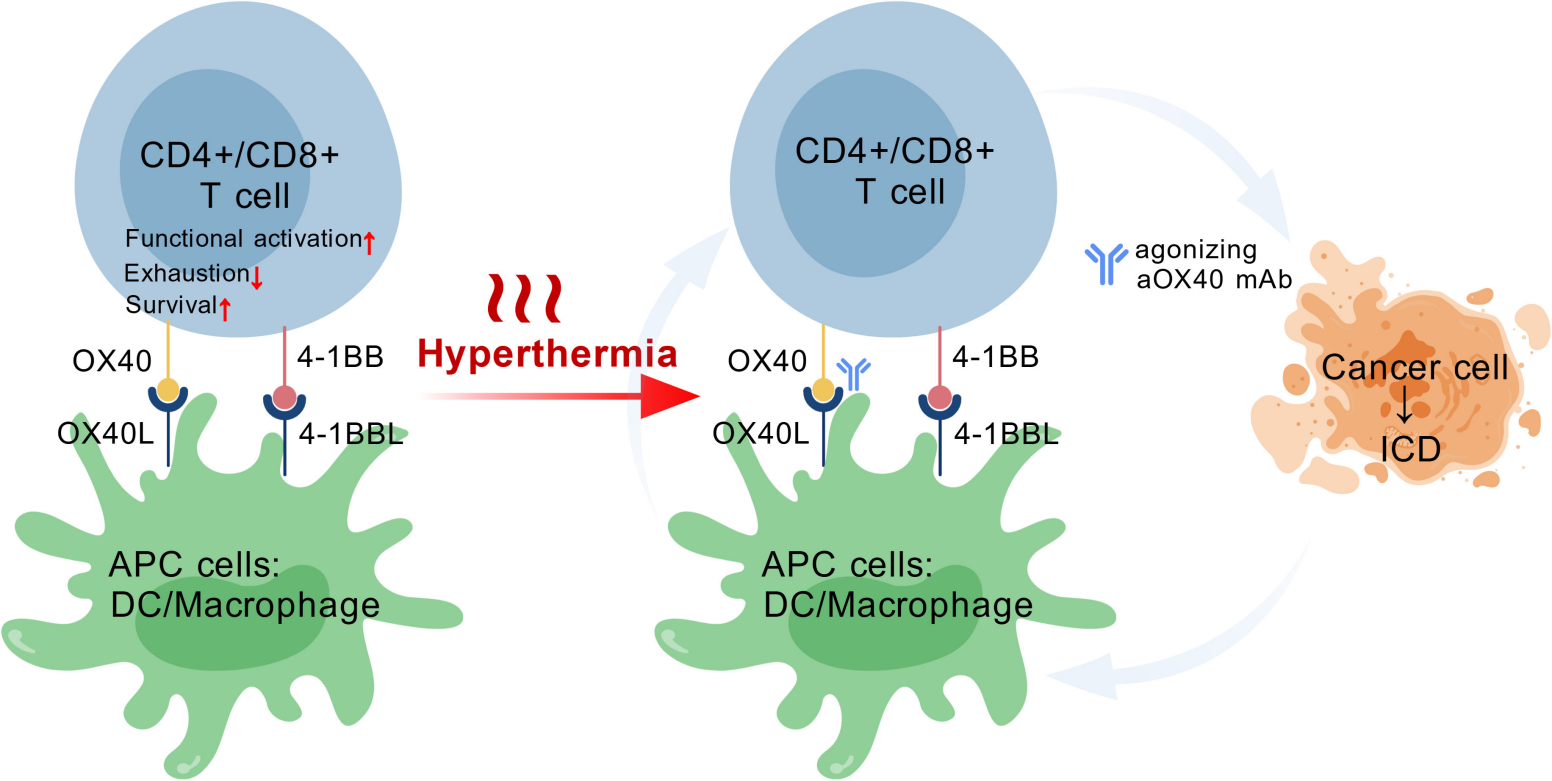
The co-stimulatory molecules OX40 and 4-1BB contribute to the functional activation and enhanced survival of CD4+ and CD8+ T cells. Their respective ligands, OX40L and 4-1BBL, are expressed on antigen-presenting cells (APCs). Hyperthermia therapy has been shown to induce immunogenic cell death (ICD) of cancer cells and promote the infiltration of CD4+ and CD8+ T cells into the tumor microenvironment.Consequently, hyperthermia may synergize with agonistic anti-OX40 and anti-4-1BB monoclonal antibodies by increasing the T cell populations.

In summary, hyperthermia and agonistic anti-OX40 mAb could synergize with each other through the following mechanism: While anti-OX40 mAb is known to promote expansion of CD4+ and CD8+ T cells, hyperthermia is capable of turning the TiME from “cold” to “hot” by improving DC maturation, increasing M1 polarization of macrophages and further amplifying the T cell-mediated anti-tumor response. An explanatory diagram is provided in **Figure 4**.

### 3.3 4-1BB

4-1BB, other known as CD137, is another T cell costimulatory molecule that regulates T cell activation (95, 96). Similar to OX40, 4-1BB is inducibly expressed on the surface of activated CD4+ and CD8+ T cells, NK cells, NKT cells, DCs and other myeloid cells (96). The ligand for 4-1BB is 4-1BBL, which is highly expressed on a variety of APCs, including dendritic cells, macrophages and B cells (96). 4-1BB activation has been suggested to boost full function of CD4+ T helper cells, prolong survival of CD8+ cytotoxic T cells and inhibit activity of Foxp3+ T-regs (97–99).

Ectopic expression of 4-1BBL (and other checkpoint proteins) has been reported in different cancer cells after heat exposure, including glioblastoma cells (~2-fold increase) (86) and breast cancer cells (~1.8-fold increase) (88). Increased expression of the immune checkpoint proteins after hyperthermia is likely to be induced by cell stress response (86), because addition of radiotherapy was found to further enhance such increase and inhibitors of the stress mediators (such as HSP70) has been found to attenuate such increase (86, 88, 89).

Combination therapy using hyperthermia and agonistic 4-1BB mAb has been reported in some recent studies. Balakrishnan PB and coworkers designed Prussian blue-based nanoparticles (PBNP) for photothermal therapy and combined PTT with anti-4-1BB antibody therapy to treat orthotopic murine SM1 melanoma (100). Their *in vitro* study found that PTT (80 °C) induced ICD (increased release of ATP, HMGB1 and calreticulin) of tumor cells in a thermal dose-dependent manner. They also observed increased T cell activation (upregulated CD69 and CD25 expression) when T cells being co-cultured with PTT-treated tumor cells. Their *in vivo* study found that PBNP-PTT caused the treated tumor to regress and addition of agonistic 4-1BB mAb further induced distal (abscopal) tumors undergoing growth inhibition. The authors observed that the combination therapy significantly increased the numbers of CD8+ T cells, activated CD4+ T cells and matured DC in TiME, and activated T cell-mediated immune memory. In another study, Medina JA et al. (from the same group) synthesized a “2-in-1” nanomaterial by conjugating PBNP with α4-1BB mAb and observed similar cancer-inhibitory effect on SM1 melanoma through immune activation (101).

Li X et al. conjugated photosensitive nanomaterial (named “TURN”) with MDSC inhibitor RGX-104 and 4-1BB agonist and applied TURN-mediated PTT (heating tumors to 50 °C) to treat 4T1 breast cancer and CT26 colon cancer tumors (102). As a result, TURN-PTT was found to cause significant inhibition of tumor growth by reducing the number of infiltrating MDSCs, activating intratumoral DCs, repolarizing macrophages towards M1 subtype, and promoting infiltration and activation (CD44+) of CD4+ and CD8+ T cells. Interestingly, the authors observed that even without RGX-104 and 4-1BB agonist, PTT by itself also caused moderate reduction of MDSCs and elevation of DCs, suggesting that hyperthermia is capable of restructuring TiME.

Together, current evidence suggests that hyperthermia and anti-4-1BB mAb may have synergistic potential to be used in cancer therapy. By promoting CD4+ T cell functionalization and prolonging CD8+ T cell survival, anti-4-1BB mAb potentiates T cell-mediated tumor suppression, whereas on the other hand, hyperthermia creates a proinflammatory microenvironment by inducing ICD and enhancing DC/macrophage- mediated innate immunity and chemoattraction of T cells (shown in **Figure 4**).

## 4 Discussion

While hyperthermia has been applied as an adjunctive therapy with radiotherapy and chemotherapy (103), some recent preclinical studies shed light on its potential to generate synergistic effect with certain immunotherapy reagents, as being discussed in this review. Obviously, there remains a long way for translation of preclinical results to clinical practice, owing to many crucial disparities between human cancers and mouse tumor models (104–106). For instance, the vast majority of human cancers are highly heterogenous and the clonal heterogenicity is naturally and tightly associated with intrinsic and acquired resistance to cancer therapies, including immunotherapy (104, 105). Most mouse tumor models, especially the cell-line-inoculated subcutaneous tumor model, are considerably homogeneous and are very likely to yield over-optimistic positive results in onco-immunology research. In addition, most human cancers, except for those closely related to tobacco smoking, have relatively low tumor mutational burden (105). Certain mouse tumor cell lines, such as Pan02 pancreatic cancer cells and CT26 colon cancer cells, were initially cultivated from mutagen-induced mouse tumors with much higher tumor mutational burden and are unsurprisingly expected to be susceptible to immunotherapy. To date, there remains no published results from randomized studies showing clear additional benefit from combining immunotherapy with hyperthermia. At this time, those encouraging results from preclinical studies should be cautiously considered a good starting point for prospective clinical studies. It may be useful to learn from these models about how to re-establish anti-tumor response from multi-modal approaches, such as hyperthermia and immune checkpoint targeting agents.

There remains a lack of consensus explaining how exactly hyperthermia affects the immune system. Obviously, different temperature used for hyperthermia treatment may generate different biological effects. Many studies discussed in our review employed nanomaterial-assisted magnetic or photothermal hyperthermia that reaches relative high temperatures above 50-60 °C, which partially or completely ablated the treated tumors (25, 26, 55, 61, 63, 67, 92, 93, 100, 102). It is believed that damaged cancer cells releases tumor specific antigens (TSAs), either freely, or in a format of HSP-Ag complex (78, 79), or being carried by dendritic cells, to the circulation to trigger anti-tumor immune response in the tumor draining lymph nodes (TDLN) and the spleen (25, 36, 55, 67). The immunogenic cell death (ICD) is also suggested to promote M1 polarization, DC maturation and CD8+ T cell infiltration in the local TiME (67, 85, 92, 100). Interestingly, in some studies, the increased T cells after tumor ablation was found to overexpress PD-1, Tim-3 and TIGIT immune checkpoint molecules (25, 55), which could be associated with a negative feedback induced by systemic IFN-γ response (107, 108). It is important to clarify whether the immune activation effect of hyperthermia is transient or durable, and understand how the positive and negative immune checkpoints (e.g. PD-1, Tim-3, TIGIT and OX40, 4-IBB) in circulating T cells, in TILs and in T cells localized in TDLN and spleen, dynamically change over time after tumor ablation. It is also important to emphasize that these nanotechnology-driven tumor ablation models should be carefully evaluated when being compared with realistic clinical practice.

Some other studies discussed in our review employed relatively lower temperature ranging from 40 to 45 °C for hyperthermia treatment conducted by water-bath, FUS and PTT methods (56, 80, 85, 94). In these studies, increased IL2 production, CD8+ T cell number and M1 polarization were observed after mild hyperthermia, which observations seem similar with those of tumor ablation. It is necessary to discriminate the immune-regulatory effects of high-temperature and mild-temperature hyperthermia, which are distinct for their mechanism(s) of action. While high temperature treatment promotes TSA release, the mild-temperature hyperthermia exerts more locoregional effects on immune signaling. It is also necessary to clarify what are the unique immune-regulatory effects of hyperthermia different from the other ICD-inducing approaches, such as radiotherapy, certain chemotherapy drugs (e.g doxorubicin, cisplatin, paclitaxel) and targeted therapies (e.g. PARP inhibitors) (109, 110), before applying hyperthermia in prospective immunotherapy- related clinical studies.

The studies discussed in our review involve four immune checkpoints/co-stimulatory (Tim-3, TIGIT, OX40 and 4-1BB) on T cells and two checkpoints (CD40 and CD47) on antigen presentation cells. While research interest in T cell-mediated immunity remains high, negative feedback loop to repress T cell immune response has been reported. For example, activated CD8+ T cells and NK cells secret IFN-γ that kills tumor cells but meanwhile induces PD-L1 expression in tumor and stromal cells through STAT1 signaling (107, 108, 111). Increased production of TGFβ and IL10 immunosuppressive cytokines has also been observed in immunotherapy of various cancers (112, 113). Alternation of the immuotherapeutic drugs targeting different checkpoints has been purposed to help to overcome the problem of immunotherapy resistance (114). Combination of ICIs with certain ICD-inducing approaches (109, 110), such as hyperthermia, might also help to enhance the therapeutic efficacy. Moreover, there are growing interest in targeting the other checkpoint/co-stimulatory molecules on dendritic cells and/or macrophages (e.g. CD47 and CD40), which would provide additional choices for future immunotherapy.

Recent evidence suggests that hyperthermia therapy is more likely to generate beneficial effects in the immunological “hot” tumors. According to Issels RD et al’s retrospective analysis of EORTC 62961-ESHO 95 clinical trial (localized high-risk soft tissue sarcoma receiving chemotherapy + hyperthermia *versus* chemotherapy only) (106), those patients whose tumors contained higher number of CD8+ T cell significantly benefited from additional hyperthermia therapy, showing longer local progression-free survival, disease free survival and overall survival, whereas individuals with lower number of CD8+ T cells showed no clear benefits from additional hyperthermia therapy (115). Therefore, hyperthermia appears to prefer a proinflammatory environment rich in tumor infiltrating lymphocytes (TILs) to exert full function, and yet more clinical trials are needed to strengthen this conclusion.

Benefiting from the development of humanized mice for immuno-oncology research (116) and organoid-based co-culture technique (115, 117), the synergistic effects between hyperthermia and immunotherapeutic agents should be more precisely and effectively evaluated using these novel approaches, eventually making it possible to perform test on the individual level.

## References

[1] Bray F Laversanne M Sung H Ferlay J Siegel RL Soerjomataram I . Global cancer statistics 2022: GLOBOCAN estimates of incidence and mortality worldwide for 36 cancers in 185 countries. CA Cancer J Clin. (2024) 74:229–63. doi: 10.3322/caac.21834. 38572751

[2] Dolgin E . The future of precision cancer therapy might be to try everything. Nature. (2024) 626:470–3. doi: 10.1038/d41586-024-00392-2. 38356072

[3] Zhu W Pan S Zhang J Xu J Zhang R Zhang Y . The role of hyperthermia in the treatment of tumor. Crit Rev Oncol Hematol. (2024) 204:104541. doi: 10.1016/j.critrevonc.2024.104541. 39461607

[4] Srinivasan ES Sankey EW Grabowski MM Chongsathidkiet P Fecci PE . The intersection between immunotherapy and laser interstitial thermal therapy: a multipronged future of neuro-oncology. Int J Hyperthermia. (2020) 37:27–34. doi: 10.1080/02656736.2020.1746413. 32672126 PMC11229985

[5] Carter TJ Agliardi G Lin FY Ellis M Jones C Robson M . Potential of magnetic hyperthermia to stimulate localized immune activation. Small. (2021) 17:e2005241. doi: 10.1002/smll.202005241. 33734595

[6] Adnan A Muñoz NM Prakash P Habibollahi P Cressman ENK Sheth RA . Hyperthermia and tumor immunity. Cancers (Basel). (2021) 13:2507. doi: 10.3390/cancers13112507. 34063752 PMC8196672

[7] Appleton E Hassan J Chan Wah Hak C Sivamanoharan N Wilkins A Samson A . Kickstarting immunity in cold tumours: Localised tumour therapy combinations with immune checkpoint blockade. Front Immunol. (2021) 12:754436. doi: 10.3389/fimmu.2021.754436. 34733287 PMC8558396

[8] Takeda T Yamada D Amano S Takeda T Kubota Y Kubota A . Hyperthermia enhances antitumor effects of immune checkpoint inhibitors and immune cell therapy. Gan To Kagaku Ryoho. (2024) 51:1053–5. 39571994

[9] Tang J Yu JX Hubbard-Lucey VM Neftelinov ST Hodge JP Lin Y . The clinical trial landscape for PD1/PDL1 immune checkpoint inhibitors. Nat Rev Drug Discov. (2018) 17:854–5. doi: 10.1038/nrd.2018.210. 30482962

[10] Doroshow DB Bhalla S Beasley MB Sholl LM Kerr KM Gnjatic S . PD-L1 as a biomarker of response to immune-checkpoint inhibitors. Nat Rev Clin Oncol. (2021) 18:345–62. doi: 10.1038/s41571-021-00473-5. 33580222

[11] Anandappa AJ Wu CJ Ott PA . Directing traffic: How to effectively drive T cells into tumors. Cancer Discov. (2020) 10:185–97. doi: 10.1158/2159-8290.CD-19-0790. 31974169 PMC7007384

[12] Hellmann MD Friedman CF Wolchok JD . Combinatorial cancer immunotherapies. Adv Immunol. (2016) 130:251–77. doi: 10.1016/bs.ai.2015.12.005. 26923003

[13] Morad G Helmink BA Sharma P Wargo JA . Hallmarks of response, resistance, and toxicity to immune checkpoint blockade. Cell. (2021) 184:5309–37. doi: 10.1016/j.cell.2021.09.020. 34624224 PMC8767569

[14] Kiaie SH Salehi-Shadkami H Sanaei MJ Azizi M Shokrollahi Barough M Nasr MS . Nano-immunotherapy: overcoming delivery challenge of immune checkpoint therapy. J Nanobiotechnol. (2023) 21:339. doi: 10.1186/s12951-023-02083-y. 37735656 PMC10512572

[15] Xiao J Shao S Deng Y Wang D Liu Y He S . Prolonged interval hypofractionated radiotherapy facilitates better antitumor immunity. Radiother Oncol. (2025) 203:110664. doi: 10.1016/j.radonc.2024.110664. 39647530

[16] Weichselbaum RR Liang H Deng L Fu YX . Radiotherapy and immunotherapy: a beneficial liaison? Nat Rev Clin Oncol. (2017) 13:2507:365–79. doi: 10.1038/nrclinonc.2016.211, PMID: 28094262

[17] Mallory M Gogineni E Jones GC Greer L Simone CB . Therapeutic hyperthermia: The old, the new, and the upcoming. Crit Rev Oncology/Hematol. (2016) 97:56–64. doi: 10.1016/j.critrevonc.2015.08.003. 26315383

[18] Crezee J Franken NAP Oei AL . Hyperthermia-based anti-cancer treatments. Cancers (Basel). (2021) 13:1240. doi: 10.3390/cancers13061240. 33808948 PMC7999567

[19] Yu X Harden K Gonzalez LC Francesco M Chiang E Irving B . The surface protein TIGIT suppresses T cell activation by promoting the generation of mature immunoregulatory dendritic cells. Nat Immunol. (2009) 10:48–57. doi: 10.1038/ni.1674. 19011627

[20] Chauvin JM Zarour HM . TIGIT in cancer immunotherapy. J Immunother Cancer. (2020) 8:e000957. doi: 10.1136/jitc-2020-000957. 32900861 PMC7477968

[21] Raphael I Kumar R McCarl LH Shoger K Wang L Sandlesh P . TIGIT and PD-1 immune checkpoint pathways are associated with patient outcome and anti-tumor immunity in glioblastoma. Front Immunol. (2021) 12:637146. doi: 10.3389/fimmu.2021.637146. 34025646 PMC8137816

[22] Meng F Li L Lu F Yue J Liu Z Zhang W . Overexpression of TIGIT in NK and T cells contributes to tumor immune escape in myelodysplastic syndromes. Front Oncol. (2020) 10:1595. doi: 10.3389/fonc.2020.01595. 32903786 PMC7438899

[23] Chauvin JM Pagliano O Fourcade J Sun Z Wang H Sander C . TIGIT and PD-1 impair tumor antigen-specific CD8^+^ T cells in melanoma patients. J Clin Invest. (2015) 125:2046–58. doi: 10.1172/JCI80445. 25866972 PMC4463210

[24] Johnston RJ Comps-Agrar L Hackney J Yu X Huseni M Yang Y . The immunoreceptor TIGIT regulates antitumor and antiviral CD8+ T cell effector function. Cancer Cell. (2014) 26:923–37. doi: 10.1016/j.ccell.2014.10.018. 25465800

[25] Chen Y Huang H Li Y Xiao W Liu Y Chen R . TIGIT blockade exerts synergistic effects on microwave ablation against cancer. Front Immunol. (2022) 13:832230. doi: 10.3389/fimmu.2022.832230. 35320940 PMC8935077

[26] Wang X Wang Y Zhao Q Feng Z Liu Q Wang G . An innovative approach to overcoming PD-1 resistance: Combined TIGIT blockade with nanophotothermal therapy. Appl Mater Today. (2024) 41:102437. doi: 10.1016/j.apmt.2024.102437. 41783259

[27] Niebel D Fröhlich A Zarbl R Fietz S de Vos L Vogt TJ . DNA methylation regulates TIGIT expression within the melanoma microenvironment, is prognostic for overall survival, and predicts progression-free survival in patients treated with anti-PD-1 immunotherapy. Clin Epigenet. (2022) 14:50. doi: 10.1186/s13148-022-01270-2. 35410311 PMC9004005

[28] Li Z Deng J Sun J Ma Y . Hyperthermia targeting the tumor microenvironment facilitates immune checkpoint inhibitors. Front Immunol. (2020) 11:595207. doi: 10.3389/fimmu.2020.595207. 33240283 PMC7680736

[29] González FE Chernobrovkin A Pereda C García-Salum T Tittarelli A López MN . Proteomic identification of heat shock-induced danger signals in a melanoma cell lysate used in dendritic cell-based cancer immunotherapy. J Immunol Res. (2018) 2018:3982942. doi: 10.1155/2018/3982942. 29744371 PMC5878886

[30] Bozaykut P Sozen E Kaga E Ece A Ozaltin E Bergquist J . HSP70 inhibition leads to the activation of proteasomal system under mild hyperthermia conditions in young and senescent fibroblasts. Oxid Med Cell Longev. (2020) 2020:9369524. doi: 10.1155/2020/9369524. 32190179 PMC7064868

[31] Krysko DV Garg AD Kaczmarek A Krysko O Agostinis P Vandenabeele P . Immunogenic cell death and DAMPs in cancer therapy. Nat Rev Cancer. (2012) 12:860–75. doi: 10.1038/nrc3380. 23151605

[32] Sweeney EE Cano-Mejia J Fernandes R . Photothermal therapy generates a thermal window of immunogenic cell death in neuroblastoma. Small. (2018) 14:e1800678. doi: 10.1002/smll.201800678. 29665282

[33] Ceelen W Demuytere J de Hingh I . Hyperthermic intraperitoneal chemotherapy: a critical review. Cancers (Basel). (2021) 13:3114. doi: 10.3390/cancers13133114. 34206563 PMC8268659

[34] Dynlacht JR Story MD Zhu WG Danner J . Lamin B is a prompt heat shock protein. J Cell Physiol. (1999) 178:28–34. doi: 10.1002/(SICI)1097-4652(199901)178:1<28::AID-JCP4>3.0.CO;2-K 9886487

[35] Nytko KJ Thumser-Henner P Russo G Weyland MS Rohrer Bley C . Role of HSP70 in response to (thermo)radiotherapy: analysis of gene expression in canine osteosarcoma cells by RNA-seq. Sci Rep. (2020) 10:12779. doi: 10.1038/s41598-020-69619-2. 32728031 PMC7391659

[36] Abreu MM Chocron AF Smadja DM . From cold to hot: mechanisms of hyperthermia in modulating tumor immunology for enhanced immunotherapy. Front Immunol. (2025) 16:1487296. doi: 10.3389/fimmu.2025.1487296. 40092992 PMC11906415

[37] Mahmood J Shukla HD Soman S Samanta S Singh P Kamlapurkar S . Immunotherapy, radiotherapy, and hyperthermia: a combined therapeutic approach in pancreatic cancer treatment. Cancers (Basel). (2018) 10:469. doi: 10.3390/cancers10120469. 30486519 PMC6316720

[38] Zeng L Liao Q Zhao Q Jiang S Yang X Tang H . Raltitrexed as a synergistic hyperthermia chemotherapy drug screened in patient-derived colorectal cancer organoids. Cancer Biol Med. (2021) 18:750–62. doi: 10.20892/j.issn.2095-3941.2020.0566. 33710819 PMC8330527

[39] Hietanen T Kapanen M Kellokumpu-Lehtinen PL . Restoring natural killer cell cytotoxicity after hyperthermia alone or combined with radiotherapy. Anticancer Res. (2016) 36:555–63. 26851009

[40] Anderson AC . Tim-3, a negative regulator of anti-tumor immunity. Curr Opin Immunol. (2012) 24:213–6. doi: 10.1016/j.coi.2011.12.005. 22226204

[41] Andrzejczak A Tupikowski K Tomkiewicz A Małkiewicz B Ptaszkowski K Domin A . The variations' in genes encoding TIM-3 and its ligand, Galectin-9, influence on ccRCC risk and prognosis. Int J Mol Sci. (2023) 24:2042. doi: 10.3390/ijms24032042. 36768365 PMC9917084

[42] Das M Zhu C Kuchroo VK . Tim-3 and its role in regulating anti-tumor immunity. Immunol Rev. (2017) 276:97–111. doi: 10.1111/imr.12520. 28258697 PMC5512889

[43] Nersesian S Carter EB Lee SN Westhaver LP Boudreau JE . Killer instincts: natural killer cells as multifactorial cancer immunotherapy. Front Immunol. (2023) 14:1269614. doi: 10.3389/fimmu.2023.1269614. 38090565 PMC10715270

[44] Yang RY Rabinovich GA Liu FT . Galectins: structure, function and therapeutic potential. Expert Rev Mol Med. (2008) 10:e17. doi: 10.1017/S1462399408000719. 18549522

[45] Yasinska IM Sakhnevych SS Pavlova L Teo Hansen Selnø A Teuscher Abeleira AM Benlaouer O . The Tim-3-Galectin-9 pathway and its regulatory mechanisms in human breast cancer. Front Immunol. (2019) 10:1594. doi: 10.3389/fimmu.2019.01594. 31354733 PMC6637653

[46] Hafler DA Kuchroo V . TIMs: central regulators of immune responses. J Exp Med. (2008) 205:2699–701. doi: 10.1084/jem.20082429. 19015312 PMC2585854

[47] Jin HT Anderson AC Tan WG West EE Ha SJ Araki K . Cooperation of Tim-3 and PD-1 in CD8 T-cell exhaustion during chronic viral infection. Proc Natl Acad Sci USA. (2010) 107:14733–8. doi: 10.1073/pnas.1009731107. 20679213 PMC2930455

[48] Nagahara K Arikawa T Oomizu S Kontani K Nobumoto A Tateno H . Galectin-9 increases Tim-3+ dendritic cells and CD8+ T cells and enhances antitumor immunity via galectin-9-Tim-3 interactions. J Immunol. (2008) 181:7660–9. doi: 10.4049/jimmunol.181.11.7660. 19017954 PMC5886706

[49] Kim WM Huang YH Gandhi A Blumberg RS . CEACAM1 structure and function in immunity and its therapeutic implications. Semin Immunol. (2019) 42:101296. doi: 10.1016/j.smim.2019.101296. 31604530 PMC6814268

[50] de Mingo Pulido Á Hänggi K Celias DP Gardner A Li J Batista-Bittencourt B . The inhibitory receptor TIM-3 limits activation of the cGAS-STING pathway in intra-tumoral dendritic cells by suppressing extracellular DNA uptake. Immunity. (2021) 54:1154–1167.e1157. doi: 10.1016/j.immuni.2021.04.019. 33979578 PMC8192496

[51] Nagata S . Apoptosis and clearance of apoptotic cells. Annu Rev Immunol. (2018) 36:489–517. doi: 10.1146/annurev-immunol-042617-053010. 29400998

[52] Wang W Wu S Cen Z Zhang Y Chen Y Huang Y . Mobilizing phospholipids on tumor plasma membrane implicates phosphatidylserine externalization blockade for cancer immunotherapy. Cell Rep. (2022) 41:111582. doi: 10.1016/j.celrep.2022.111582. 36323258 PMC9671066

[53] Smith CM Li A Krishnamurthy N Lemmon MA . Phosphatidylserine binding directly regulates TIM-3 function. Biochem J. (2021) 478:3331–49. doi: 10.1042/BCJ20210425. 34435619 PMC8454703

[54] Yang X Li M Qin X Tan S Du L Ma C . Photophosphatidylserine guides natural killer cell photoimmunotherapy via Tim-3. J Am Chem Soc. (2022) 144:3863–74. doi: 10.1021/jacs.1c11498. 35226805

[55] Huang TY Huang GL Zhang CY Zhuang BW Liu BX Su LY . Supramolecular photothermal nanomedicine mediated distant tumor inhibition via PD-1 and TIM-3 blockage. Front Chem. (2020) 8:1. doi: 10.3389/fchem.2020.00001. 32117862 PMC7034522

[56] Ren H Zhang Y Huang W Xu H He W Hao N . Tumor-targeted nanodrug FSGG/siGal-9 for transdermal photothermal immunotherapy of melanoma. Commun Biol. (2024) 7:188. doi: 10.1038/s42003-024-05891-6. 38366083 PMC10873409

[57] Shi L Chen L Wu C Zhu Y Xu B Zheng X . PD-1 blockade boosts radiofrequency ablation-elicited adaptive immune responses against tumor. Clin Cancer Res. (2016) 22:1173–84. doi: 10.1158/1078-0432.CCR-15-1352. 26933175 PMC4780056

[58] Feng Y Huang C Wang Y Chen J . SIRPα: A key player in innate immunity. Eur J Immunol. (2023) 53:e2350375. doi: 10.1002/eji.202350375. 37672390

[59] Matozaki T Murata Y Okazawa H Ohnishi H . Functions and molecular mechanisms of the CD47–SIRPα signalling pathway. Trends Cell Biol. (2009) 19:72–80. doi: 10.1016/j.tcb.2008.12.001. 19144521

[60] Jiang Z Sun H Yu J Tian W Song Y . Targeting CD47 for cancer immunotherapy. J Hematol Oncol. (2021) 14:180. doi: 10.1186/s13045-021-01197-w. 34717705 PMC8557524

[61] Wu CC Yang YC Hsu YT Wu TC Hung CF Huang JT . Nanoparticle-induced intraperitoneal hyperthermia and targeted photoablation in treating ovarian cancer. Oncotarget. (2015) 6:26861–75. doi: 10.18632/oncotarget.4766. 26318039 PMC4694958

[62] Rezaei G Habibi-Anbouhi M Mahmoudi M Azadmanesh K Moradi-Kalbolandi S Behdani M . Development of anti-CD47 single-chain variable fragment targeted magnetic nanoparticles for treatment of human bladder cancer. Nanomedicine (Lond). (2017) 12:597–613. doi: 10.2217/nnm-2016-0302. 28186432

[63] Ji X Qian X Luo G Yang W Huang W Lei Z . Engineered macrophage nanoparticles enhance microwave ablation efficacy in osteosarcoma via targeting the CD47-SIRPα axis: A novel biomimetic immunotherapeutic approach. Bioact Mater. (2025) 47:248–65. doi: 10.1016/j.bioactmat.2025.01.012. 39925711 PMC11803168

[64] Mouratidis PXE Ter Haar G . HSP90 inhibition acts synergistically with heat to induce a pro-immunogenic form of cell death in colon cancer cells. Int J Hyperthermia. (2021) 38:1443–56. doi: 10.1080/02656736.2021.1983036. 34612127

[65] Adkins I Sadilkova L Hradilova N Tomala J Kovar M Spisek R . Severe, but not mild heat-shock treatment induces immunogenic cell death in cancer cells. Oncoimmunology. (2017) 6:e1311433. doi: 10.1080/2162402X.2017.1311433. 28638734 PMC5467989

[66] Wang S Jiao W Yan B Liu X Tang Q Zhang Y . Intracellular magnetic hyperthermia enables concurrent down-regulation of CD47 and SIRPα to potentiate antitumor immunity. Nano Lett. (2024) 24:2894–903. doi: 10.1021/acs.nanolett.4c00003. 38407042

[67] Chang M Hou Z Jin D Zhou J Wang M Wang M . Colorectal tumor microenvironment-activated bio-decomposable and metabolizable Cu(2) O@CaCO(3) nanocomposites for synergistic oncotherapy. Adv Mater. (2020) 32:e2004647. doi: 10.1002/adma.202004647, PMID: 32945002

[68] Zanganeh S Hutter G Spitler R Lenkov O Mahmoudi M Shaw A . Iron oxide nanoparticles inhibit tumour growth by inducing pro-inflammatory macrophage polarization in tumour tissues. Nat Nanotechnol. (2016) 11:986–94. doi: 10.1038/nnano.2016.168. 27668795 PMC5198777

[69] Liu X Pu Y Cron K Deng L Kline J Frazier WA . CD47 blockade triggers T cell-mediated destruction of immunogenic tumors. Nat Med. (2015) 21:1209–15. doi: 10.1038/nm.3931. 26322579 PMC4598283

[70] Frey B Weiss EM Rubner Y Wunderlich R Ott OJ Sauer R . Old and new facts about hyperthermia-induced modulations of the immune system. Int J Hyperthermia. (2012) 28:528–42. doi: 10.3109/02656736.2012.677933. 22690925

[71] Elgueta R Benson MJ de Vries VC Wasiuk A Guo Y Noelle RJ . Molecular mechanism and function of CD40/CD40L engagement in the immune system. Immunol Rev. (2009) 229:152–72. doi: 10.1111/j.1600-065X.2009.00782.x. 19426221 PMC3826168

[72] Bullock TNJ . CD40 stimulation as a molecular adjuvant for cancer vaccines and other immunotherapies. Cell Mol Immunol. (2022) 19:14–22. doi: 10.1038/s41423-021-00734-4. 34282297 PMC8752810

[73] Akbulut Z Aru B Aydın F Yanıkkaya Demirel G . Immune checkpoint inhibitors in the treatment of hepatocellular carcinoma. Front Immunol. (2024) 15:1379622. doi: 10.3389/fimmu.2024.1379622. 38638433 PMC11024234

[74] Luri-Rey C Teijeira Á Wculek SK de Andrea C Herrero C Lopez-Janeiro A . Cross-priming in cancer immunology and immunotherapy. Nat Rev Cancer. (2025) 25:249–73. doi: 10.1038/s41568-024-00785-5. 39881005

[75] Tournier JN Hellmann AQ Lesca G Jouan A Drouet E Mathieu J . Fever-like thermal conditions regulate the activation of maturing dendritic cells. J Leukoc Biol. (2003) 73:493–501. doi: 10.1189/jlb.1002506. 12660224

[76] Basu S Srivastava PK . Fever-like temperature induces maturation of dendritic cells through induction of hsp90. Int Immunol. (2003) 15:1053–61. doi: 10.1093/intimm/dxg104. 12917257

[77] Zheng H Benjamin IJ Basu S Li Z . Heat shock factor 1-independent activation of dendritic cells by heat shock: implication for the uncoupling of heat-mediated immunoregulation from the heat shock response. Eur J Immunol. (2003) 33:1754–62. doi: 10.1002/eji.200323687. 12778494

[78] Salimu J Spary LK Al-Taei S Clayton A Mason MD Staffurth J . Cross-presentation of the oncofetal tumor antigen 5T4 from irradiated prostate cancer cells--a key role for heat-shock protein 70 and receptor CD91. Cancer Immunol Res. (2015) 3:678–88. doi: 10.1158/2326-6066.CIR-14-0079. 25678582

[79] Murshid A Gong J Calderwood SK . The role of heat shock proteins in antigen cross presentation. Front Immunol. (2012) 3:63. doi: 10.3389/fimmu.2012.00063. 22566944 PMC3342350

[80] Singh MP Sethuraman SN Ritchey J Fiering S Guha C Malayer J . In-situ vaccination using focused ultrasound heating and anti-CD-40 agonistic antibody enhances T-cell mediated local and abscopal effects in murine melanoma. Int J Hyperthermia. (2019) 36:64–73. doi: 10.1080/02656736.2019.1663280. 31795832 PMC6897315

[81] Fu Y Lin Q Zhang Z Zhang L . Therapeutic strategies for the costimulatory molecule OX40 in T-cell-mediated immunity. Acta Pharm Sin B. (2020) 10:414–33. doi: 10.1016/j.apsb.2019.08.010. 32140389 PMC7049610

[82] Thapa B Kato S Nishizaki D Miyashita H Lee S Nesline MK . OX40/OX40 ligand and its role in precision immune oncology. Cancer Metastasis Rev. (2024) 43:1001–13. doi: 10.1007/s10555-024-10184-9. 38526805 PMC11300540

[83] Willoughby J Griffiths J Tews I Cragg MS . OX40: Structure and function - what questions remain? Mol Immunol. (2017) 83:13–22. doi: 10.1016/j.molimm.2017.01.006, PMID: 28092803

[84] Watts TH Yeung KKM Yu T Lee S Eshraghisamani R . TNF/TNFR superfamily members in costimulation of T cell responses-revisited. Annu Rev Immunol. (2025) 43:113–42. doi: 10.1146/annurev-immunol-082423-040557. 39745933

[85] Mahmood J Alexander AA Samanta S Kamlapurkar S Singh P Saeed A . A combination of radiotherapy, hyperthermia, and immunotherapy inhibits pancreatic tumor growth and prolongs the survival of mice. Cancers (Basel). (2020) 12:1015. doi: 10.3390/cancers12041015. 32326142 PMC7226594

[86] Stoll E Hader M Rückert M Weissmann T Lettmaier S Putz F . Detailed *in vitro* analyses of the impact of multimodal cancer therapy with hyperthermia and radiotherapy on the immune phenotype of human glioblastoma cells. Int J Hyperthermia. (2022) 39:796–805. doi: 10.1080/02656736.2022.2080873. 35676615

[87] Ferencz B Török K Pipek O Fillinger J Csende K Lantos A . Expression patterns of novel immunotherapy targets in intermediate- and high-grade lung neuroendocrine neoplasms. Cancer Immunol Immunother. (2024) 73:114. doi: 10.1007/s00262-024-03704-7. 38693435 PMC11063022

[88] Hader M Savcigil DP Rosin A Ponfick P Gekle S Wadepohl M . Differences of the immune phenotype of breast cancer cells after ex vivo hyperthermia by warm-water or microwave radiation in a closed-loop system alone or in combination with radiotherapy. Cancers (Basel). (2020) 12:1082. doi: 10.3390/cancers12051082. 32349284 PMC7281749

[89] Szwed M Jost T Majka E Gharibkandi NA Majkowska-Pilip A Frey B . Pt-Au nanoparticles in combination with near-infrared-based hyperthermia increase the temperature and impact on the viability and immune phenotype of human hepatocellular carcinoma cells. Int J Mol Sci. (2025) 26:1574. doi: 10.3390/ijms26041574. 40004038 PMC11855494

[90] Shibahara I Saito R Zhang R Chonan M Shoji T Kanamori M . OX40 ligand expressed in glioblastoma modulates adaptive immunity depending on the microenvironment: a clue for successful immunotherapy. Mol Cancer. (2015) 14:41. doi: 10.1186/s12943-015-0307-3. 25744203 PMC4339477

[91] Lin L Hu Y Guo Z Chen J Sun P Tian H . Gene-guided OX40L anchoring to tumor cells for synergetic tumor “self-killing” immunotherapy. Bioact Mater. (2023) 25:689–700. doi: 10.1016/j.bioactmat.2022.07.008. 37056266 PMC10086763

[92] Ni W Wu J Fang H Feng Y Hu Y Lin L . Photothermal-chemotherapy enhancing tumor immunotherapy by multifunctional metal-organic framework based drug delivery system. Nano Lett. (2021) 21:7796–805. doi: 10.1021/acs.nanolett.1c02782. 34516141

[93] Chang X Wu Q Wu Y Xi X Cao J Chu H . Multifunctional Au modified Ti(3)C(2)-MXene for photothermal/enzyme dynamic/immune synergistic therapy. Nano Lett. (2022) 22:8321–30. doi: 10.1021/acs.nanolett.2c03260. 36222477

[94] Momenzadeh N Hajian S Shabankare A Ghavimi R Kabiri-Samani S Kabiri H . Photothermic therapy with cuttlefish ink-based nanoparticles in combination with anti-OX40 mAb achieve remission of triple-negative breast cancer. Int Immunopharmacol. (2023) 115:109622. doi: 10.1016/j.intimp.2022.109622. 36577156

[95] Kraehenbuehl L Weng CH Eghbali S Wolchok JD Merghoub T . Enhancing immunotherapy in cancer by targeting emerging immunomodulatory pathways. Nat Rev Clin Oncol. (2022) 19:37–50. doi: 10.1038/s41571-021-00552-7. 34580473

[96] Choi Y Shi Y Haymaker CL Naing A Ciliberto G Hajjar J . T-cell agonists in cancer immunotherapy. J Immunother Cancer. (2020) 8:e000966. doi: 10.1136/jitc-2020-000966. 33020242 PMC7537335

[97] Martinez-Forero I Azpilikueta A Bolaños-Mateo E Nistal-Villan E Palazon A Teijeira A . T cell costimulation with anti-CD137 monoclonal antibodies is mediated by K63-polyubiquitin-dependent signals from endosomes. J Immunol. (2013) 190:6694–706. doi: 10.4049/jimmunol.1203010. 23690480

[98] So T Croft M . Regulation of PI-3-kinase and Akt signaling in T lymphocytes and other cells by TNFR family molecules. Front Immunol. (2013) 4:139. doi: 10.3389/fimmu.2013.00139. 23760533 PMC3675380

[99] So T Lee SW Croft M . Immune regulation and control of regulatory T cells by OX40 and 4-1BB. Cytokine Growth Factor Rev. (2008) 19:253–62. doi: 10.1016/j.cytogfr.2008.04.003. 18508403 PMC2486494

[100] Balakrishnan PB Ledezma DK Cano-Mejia J Andricovich J Palmer E Patel VA . CD137 agonist potentiates the abscopal efficacy of nanoparticle-based photothermal therapy for melanoma. Nano Res. (2022) 15:2300–14. doi: 10.1007/s12274-021-3813-1. 36089987 PMC9455608

[101] Medina JA Ledezma DK Ghofrani J Chen J Chin SJ Balakrishnan PB . Photothermal therapy co-localized with CD137 agonism improves survival in an SM1 melanoma model without hepatotoxicity. Nanomedicine (Lond). (2024) 19:2049–64. doi: 10.1080/17435889.2024.2389770. 39225150 PMC11485692

[102] Li X Liang X Fu W Luo R Zhang M Kou X . Reversing cancer immunoediting phases with a tumor-activated and optically reinforced immunoscaffold. Bioact Mater. (2024) 35:228–41. doi: 10.1016/j.bioactmat.2024.01.026. 38333614 PMC10850754

[103] Oei AL Kok HP Oei SB Horsman MR Stalpers LJA Franken NAP . Molecular and biological rationale of hyperthermia as radio- and chemosensitizer. Adv Drug Delivery Rev. (2020) 163-164:84–97. doi: 10.1016/j.addr.2020.01.003. 31982475

[104] Laplane L Maley CC . The evolutionary theory of cancer: Challenges and potential solutions. Nat Rev Cancer. (2024) 24:718–33. doi: 10.1038/s41568-024-00734-2. 39256635 PMC11627115

[105] Martínez-Jiménez F Chowell D . Genetic immune escape in cancer: Timing and implications for treatment. Trends Cancer. (2025) 11:286–94. doi: 10.1016/j.trecan.2024.11.002. 39632211 PMC11981860

[106] Issels RD Noessner E Lindner LH Schmidt M Albertsmeier M Blay JY . Immune infiltrates in patients with localised high-risk soft tissue sarcoma treated with neoadjuvant chemotherapy without or with regional hyperthermia: A translational research program of the EORTC 62961-ESHO 95 randomised clinical trial. Eur J Cancer. (2021) 158:123–32. doi: 10.1016/j.ejca.2021.09.015. 34666214

[107] Ribas A . Adaptive immune resistance: How cancer protects from immune attack. Cancer Discov. (2015) 5:915–9. doi: 10.1158/2159-8290.CD-15-0563. 26272491 PMC4560619

[108] Pistillo MP Carosio R Banelli B Morabito A Mastracci L Ferro P . IFN-γ upregulates membranous and soluble PD-L1 in mesothelioma cells: Potential implications for the clinical response to PD-1/PD-L1 blockade. Cell Mol Immunol. (2020) 17:410–1. doi: 10.1038/s41423-019-0245-x. 31217525 PMC7109117

[109] Kroemer G Galassi C Zitvogel L Galluzzi L . Immunogenic cell stress and death. Nat Immunol. (2022) 23:487–500. doi: 10.1038/s41590-022-01132-2. 35145297

[110] Galluzzi L Guilbaud E Schmidt D Kroemer G Marincola FM . Targeting immunogenic cell stress and death for cancer therapy. Nat Rev Drug Discov. (2024) 23:445–60. doi: 10.1038/s41573-024-00920-9. 38622310 PMC11153000

[111] Ribas A Wolchok JD . Cancer immunotherapy using checkpoint blockade. Science. (2018) 359:1350–5. doi: 10.1126/science.aar4060. 29567705 PMC7391259

[112] Mariathasan S Turley SJ Nickles D Castiglioni A Yuen K Wang Y . TGFβ attenuates tumour response to PD-L1 blockade by contributing to exclusion of T cells. Nature. (2018) 554:544–8. doi: 10.1038/nature25501. 29443960 PMC6028240

[113] Lamichhane P Karyampudi L Shreeder B Krempski J Bahr D Daum J . IL10 release upon PD-1 blockade sustains immunosuppression in ovarian cancer. Cancer Res. (2017) 77:6667–78. doi: 10.1158/0008-5472.CAN-17-0740. 28993412 PMC5712245

[114] Koyama S Akbay EA Li YY Herter-Sprie GS Buczkowski KA Richards WG . Adaptive resistance to therapeutic PD-1 blockade is associated with upregulation of alternative immune checkpoints. Nat Commun. (2016) 7:10501. doi: 10.1038/ncomms10501. 26883990 PMC4757784

[115] Bar-Ephraim YE Kretzschmar K Clevers H . Organoids in immunological research. Nat Rev Immunol. (2020) 20:279–93. doi: 10.1038/s41577-019-0248-y. 31853049

[116] Chuprin J Buettner H Seedhom MO Greiner DL Keck JG Ishikawa F . Humanized mouse models for immuno-oncology research. Nat Rev Clin Oncol. (2023) 20:192–206. doi: 10.1038/s41571-022-00721-2. 36635480 PMC10593256

[117] Wang Q Yuan F Zuo X Li M . Breakthroughs and challenges of organoid models for assessing cancer immunotherapy: A cutting-edge tool for advancing personalised treatments. Cell Death Discov. (2025) 11:222. doi: 10.1038/s41420-025-02505-w. 40335487 PMC12059183

